# Entropy, complexity, and Markov diagrams for random walk cancer models

**DOI:** 10.1038/srep07558

**Published:** 2014-12-19

**Authors:** Paul K. Newton, Jeremy Mason, Brian Hurt, Kelly Bethel, Lyudmila Bazhenova, Jorge Nieva, Peter Kuhn

**Affiliations:** 1 Viterbi School of Engineering, Department of Mathematics, and Norris Comprehensive Cancer Center, University of Southern California, Los Angeles, CA 90089-1191, USA; 2 Scripps Clinic Medical Group, 10666 N. Torrey Pines Rd. MC 211C, La Jolla CA 92037; 3 UCSD Moores Cancer Center, 3855 Health Sciences Drive, La Jolla, CA, 92093; 4 Keck School of Medicine, University of Southern California, 1441 Eastlake Avenue Suite 3440, Los Angeles, CA 90033; 5 Department of Biological Sciences, University of Southern California, 3430 S. Vermont Ave, Suite 105, Los Angeles CA 90089-3301

## Abstract

The notion of entropy is used to compare the complexity associated with 12 common cancers based on metastatic tumor distribution autopsy data. We characterize power-law distributions, entropy, and Kullback-Liebler divergence associated with each primary cancer as compared with data for all cancer types aggregated. We then correlate entropy values with other measures of complexity associated with Markov chain dynamical systems models of progression. The Markov transition matrix associated with each cancer is associated with a directed graph model where nodes are anatomical locations where a metastatic tumor could develop, and edge weightings are transition probabilities of progression from site to site. The steady-state distribution corresponds to the autopsy data distribution. Entropy correlates well with the overall complexity of the reduced directed graph structure for each cancer and with a measure of systemic interconnectedness of the graph, called graph conductance. The models suggest that grouping cancers according to their entropy values, with skin, breast, kidney, and lung cancers being prototypical high entropy cancers, stomach, uterine, pancreatic and ovarian being mid-level entropy cancers, and colorectal, cervical, bladder, and prostate cancers being prototypical low entropy cancers, provides a potentially useful framework for viewing metastatic cancer in terms of predictability, complexity, and metastatic potential.

Metastatic cancer is a dynamic disease of relentlessly increasing entropy. From an initial primary tumor located at a single anatomical site (a zero-entropy state), the metastatic cascade leads to a proliferation of tumors at other sites on a timescale of months, or years in most cases, if left untreated^1^,^2^,^3^,^4^. Entropy is a quantity deeply connected with notions of complexity and predictability^5^,^6^ used primarily in the fields of information theory^7^,^8^,^9^ and statistical thermodynamics^10^. It is used to quantify the level of disorder associated with a stochastic dynamical process that has a number of sites that it can occupy^11^,^12^. Systems that can visit these sites with relatively equal probability have higher entropy (they are considered more disordered and less predictable) than systems that can only occupy a few sites with very different probabilities (considered less disordered and more predictable). In this paper we demonstrate how entropy, relative entropy^6^,^13^, and graph conductance^30^ can be used in the context of metastatic spread to quantify, compare, and co-group the 12 most prevalent cancer types worldwide. The view of cancer that we describe is based on its *dynamical* characteristics, which offers a more nuanced view than the static view of classifying only according to site of origin. To put it differently, we characterize cancers based not just on their initial conditions, but on a collection of features that are associated with their dynamical predictability throughout the course of disease progression.

To fix ideas further, suppose each anatomical site where a primary or metastatic tumor could appear is indexed by ‘i’, (i = 1,…N). Let *σ_i_* represent the probability that site ‘i’ is occupied (i.e. has a metastatic tumor), and let 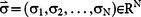 represent a probability mass distribution over a collection of potentially occupied sites, so that 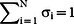, with 0 ≤ *σ_j_* ≤ 1. The level of disorder associated with the distribution 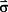 is captured by a scalar quantity *H_N_*_′_called the *entropy* of the state, a quantity that is a function both of N, and the way the probabilities are distributed among the N sites. The lowest entropy state, corresponding to the one of least disorder, would be represented by a distribution such as 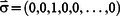, in which case *H_N_* = 0. In this distribution, state i = 3 is occupied with probability 1, making it predictively certain. Typically, this site would be the anatomical location of the primary tumor in a Stage I patient whose disease has not yet progressed. The highest entropy state, corresponding to the one of most disorder, would be represented by the uniform distribution 
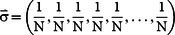
. For this uniform distribution, each site is occupied with equal probability. This distribution, which constitutes a mathematical upper bound on the entropy, represents a state of maximal disorder. It corresponds to the least predictable state. In the case where only two states are possible (N = 2), a familiar example of a maximum entropy state would be the probability of heads (H) and tails (T) when flipping a fair coin, P(H) = ½, P(T) = ½, 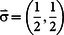. The point we want to emphasize is that associated with any specific probabilistic distribution of occupied sites (typically falling between the above two extremes, as shown, for example in Figure 1 for all cancers grouped together), is a quantitative notion of disorder, which in turn is related to the system’s predictability and complexity^5^,^6^,^11^,^12^. Since each cancer type has a different empirical metastatic tumor distribution, each will have a different entropy value and these entropy values can be thought of as convenient and simple surrogates that represent metastatic tumor complexity and disorder associated with ensemble populations of patients with a given primary cancer type.

We show in this paper how entropy is a useful metric of metastatic complexity that correlates well with features associated with the anatomical pathways of disease progression, as well as the graph conductance measuring the density of the network diagram, which in turn is associated with rates of convergence to the steady-state tumor distribution of the disease in a population of patients. We note that notions of entropy have been used fruitfully to characterize other aspects of metastatic cancer both at the genotypic and phenotypic levels^24^,^25^,^26^,^27^,^29^,^33^,^34^,^35^,^36^ but none have related it to large scale progression patterns with the goal of quantifying complexity and predictability.

## Results

### Distribution of metastatic tumors from autopsy data

**Figure 1:**
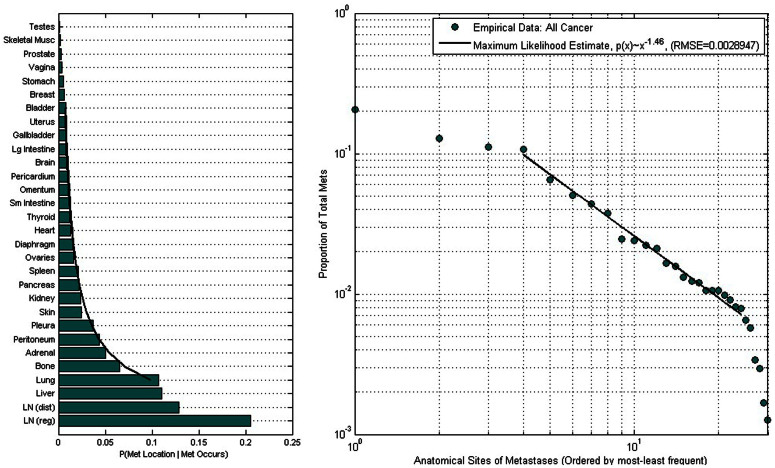
Histogram (left) of distribution of metastatic tumors over all cancer types from 3827 patients, 9484 metastatic tumors distributed over 30 anatomical sites. Data is plotted on log-log plot (right) showing power law form p(x) ~ x^−1.46^ (RMSE = 0.0028947) obtained using a maximum likelihood estimator and goodness-of-fit criteria to obtain the best range of the power law distribution^14^.

Figure 1 shows the tumor distribution for all cancers collected from 3827 untreated patients, with a total of 9484 metastatic tumors distributed over 30 distinct anatomical sites^14^. On the left we show the histograms, normalized so that the total area under the bars is one, hence the distribution represents the probability mass function associated with ‘all’ cancers. On the right we show the same data plotted on a log-log plot to more clearly bring out the fact that there is a power-law scaling range, where the distribution follows the form p(x)~x^−α^, with α = 1.46, obtained using maximum likelihood estimators, and a goodness-of-fit criterion for the optimal range over which the power-law holds^15^. We note that power-law distributions arise in many other contexts, most relevant might be the distribution of edges from nodes on the World-Wide-Web^16^. The analogy of web-surfing from site-to-site and modeling cancer progression as a random walk process from site-to-site has been used fruitfully in^17^,^18^ and is the basis for the Markov diagrams described later.

**Figure 2:**
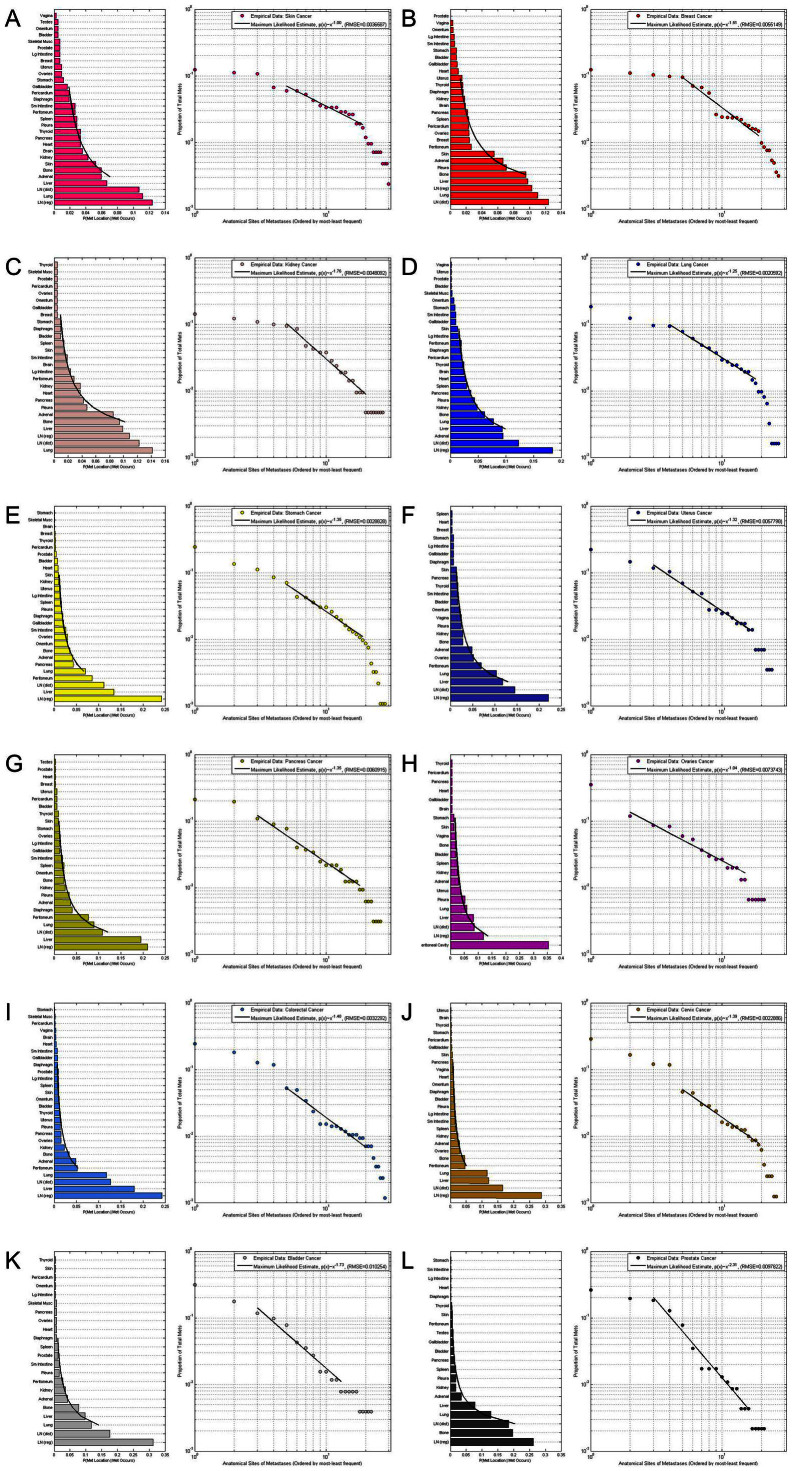
Histograms of distribution of metastatic tumors for primary cancers. Data is plotted on a log-log plot for each, showing power law form. (a) Skin cancer: 163 patients, 619 metastases, 27 anatomical sites, p(x) ~ x^−1.25^ (RMSE = 0.0036587); (b) Breast cancer: 432 patients, 2235 metastases, 28 anatomical sites, p(x) ~ x^−1.51^ (RMSE = 0.0055149); (c) Kidney cancer: 193 patients, 462 metastases, 21 anatomical sites, p(x) ~ x^−2.31^ (RMSE = 0.0048092); (d) Lung cancer: 560 patients, 859 metastases, 28 anatomical sites, p(x) ~ x^−1.46^ (RMSE = 0.0020592); (e) Stomach cancer: 109 patients, 323 metastases, 26 anatomical sites, p(x) ~ x^−1.35^ (RMSE = 0.0028828); and (f) Uterine cancer: 86 patients, 302 metastases, 26 anatomical sites, p(x) ~ x^−1.05^ (RMSE = 0.0057798). (g) Pancreatic cancer: 183 patients, 256 metastases, 22 anatomical sites, p(x) ~ x^−1.73^ (RMSE = 0.0060915); (h) Ovarian cancer: 418 patients, 806 metastases, 26 anatomical sites, p(x) ~ x^−1.39^ (RMSE = 0.0073743); (i) Colorectal cancer: 161 patients, 420 metastases, 30 anatomical sites, p(x) ~ x^−1.00^ (RMSE = 0.0032292); (j) Cervical cancer: 348 patients, 928 metastases, 28 anatomical sites, p(x) ~ x^−1.35^ (RMSE = 0.0022886); (k) Bladder cancer: 120 patients, 289 metastases, 24 anatomical sites, p(x) ~ x^−1.32^ (RMSE = 0.010254); and (l) Prostate cancer: 62 patients, 212 metastases, 26 anatomical sites, p(x) ~ x^−1.76^ (RMSE = 0.0097822).

The panels shown in Figure 2a-l break the data of Figure 1 into 12 groupings associated with 12 major primary cancer types (a. Skin; b. Breast; c. Kidney; d. Lung; e. Stomach; f. Uterine; g. Pancreatic; h. Ovarian; i. Colorectal; j. Cervical; k. Bladder; l. Prostate) and the ensemble metastatic distributions associated with each. Each of the empirical distributions shows a clear power-law range (details are described in Figure caption), each with a distinct power-law exponent and approximate range of validity.

As detailed in^17^,^18^ for our lung cancer model, these metastatic tumor distributions are used to construct Markov chain dynamical system models of cancer progression. Markov transition matrices^28^ are constructed conditioned on (i) these distributions being steady-states, and (ii) an initial ‘trial’ matrix which is used to produce converged Markov matrices for each cancer type using a stochastic iteration scheme, where the entries of the trial matrix are obtained from the data. Details are described in the Materials and Methods section and specifically for the lung cancer model in^17^,^18^.

### Metastatic entropy for 12 major cancer types

Because of well known difficulties inherent with pinning down precise values for power-law exponents^15^, we do not use their value for comparative purposes. For the purposes of quantifying the complexity associated with each primary cancer type, we calculate the entropy associated with each, given by the formula 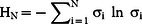, where *σ_i_* represents the proportion of metastatic tumors found at anatomical site ‘i’, for a given primary cancer type. The constraints are given by 0 ≤ σ_j_ ≤ 1, (i = 1, …, N), 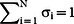. Entropy has been used in various contexts related to cancer in the literature, see for example^33^,^34^,^35^,^36^. It should be intuitively clear that an increase in complexity is associated with two distinct features associated with each of the distributions: (i) the total number of sites, N, at which metastatic tumors are found, and (ii) relatively flat distributions, meaning that the probabilities of spreading to each site are more equally probable than what a steep distribution would show. Both of these factors play an important role in the entropy values. Table 1 shows the value of the metastatic entropy for each of the 12 cancer types, as well as the ‘All Cancer’ aggregated data. The first column lists the primary cancer type, the second column lists the number of sites, N, over which the metastatic tumors are distributed, while the third column lists the metastatic entropy associated with the empirical distributions shown in Figures 1 and 2. We list the sites according to the descending values of the entropy shown in Table 1, column 3, thus skin (2.9945), breast (2.7798), kidney (2.7554), and lung (2.7454) all have entropy values higher than the value for all cancers combined (2.7136), which we use as a benchmark for comparisons. The cancer type with the lowest entropy value is prostate (2.0960), consistent with the relatively small number of sites to which it distributes (N = 21), and the relatively sharp drop in the empirical distribution shown in Figure 2l. It is useful to compare this distribution with skin, shown in Figure 2a, which has more sites to which it distributes itself (N = 30), and has a distinctly flatter distribution to those sites. For ovarian cancer, whose entropy is relatively low (2.5193), we have grouped large intestine, small intestine, diaphragm, ovary, omentum, and peritoneum all as one site which we call ‘peritoneal cavity’, due to the fact that metastases in each of these regions likely represent random spread of disease within an anatomically connected region, as opposed to hematogenously seeded metastases. To get an idea of the robustness of these ‘Autopsy Entropy’ values, we show in column 4 ‘Sample Entropy’ values, with +/− standard deviations. These are computed using the Markov transition matrices for each cancer type to produce artificial sample populations (100 sample populations for each cancer type, each with the same number of patients as in the autopsy data) with the same statistical characteristics as the autopsy populations (i.e. same total number of patients, same number of metastatic tumors and tumor distributions). We use these sample populations to obtain standard deviation values.

### Relative-entropy between each primary cancer type and the aggregate entropy associated with all cancers

Columns 5 and 6 in Table 1 show the Kullback-Liebler divergence^8^ between each cancer type and the all cancer category. We use Q as the all cancer distribution, while P is the distribution associated with each specific cancer type. While the value of entropy shown in column 3 is independent of the ordering in which the sites are listed, the K-L divergence is not. In column 6 we calculate this quantity using the P distribution and the Q distribution arranged in decreasing order in each case. As Table 1 column 5 indicates, the K-L divergence between prostate and ‘All’ is the highest (0.1620), indicating that its shape is most different from the all cancer category. By contrast, stomach cancer has the smallest K-L divergence from the all cancer group (0.0213), making it in this sense, the most similar to the aggregate.

**Figure 3:**
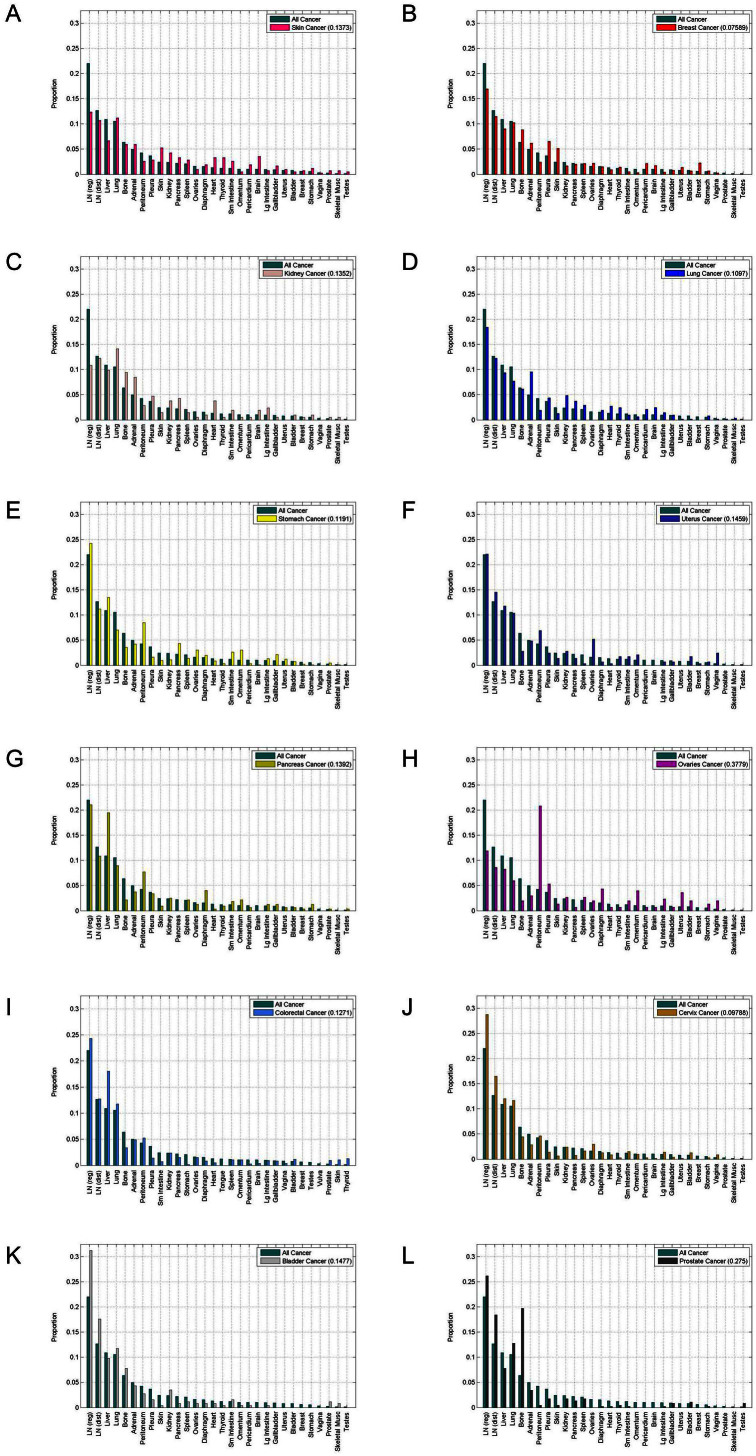
Site specific histograms of distribution of metastatic tumors for primary cancers compared with distribution of all cancer. Data is plotted according to sites in descending order corresponding to the all cancer distribution. (a) Skin cancer; (b) Breast cancer; (c) Kidney cancer; (d) Lung cancer; (e) Stomach cancer; and (f) Uterine cancer. (g) Pancreatic cancer; (h) Ovarian cancer; (i) Colorectal cancer; (j) Cervical cancer; (k) Bladder cancer; and (l) Prostate cancer.

**Figure 4:**
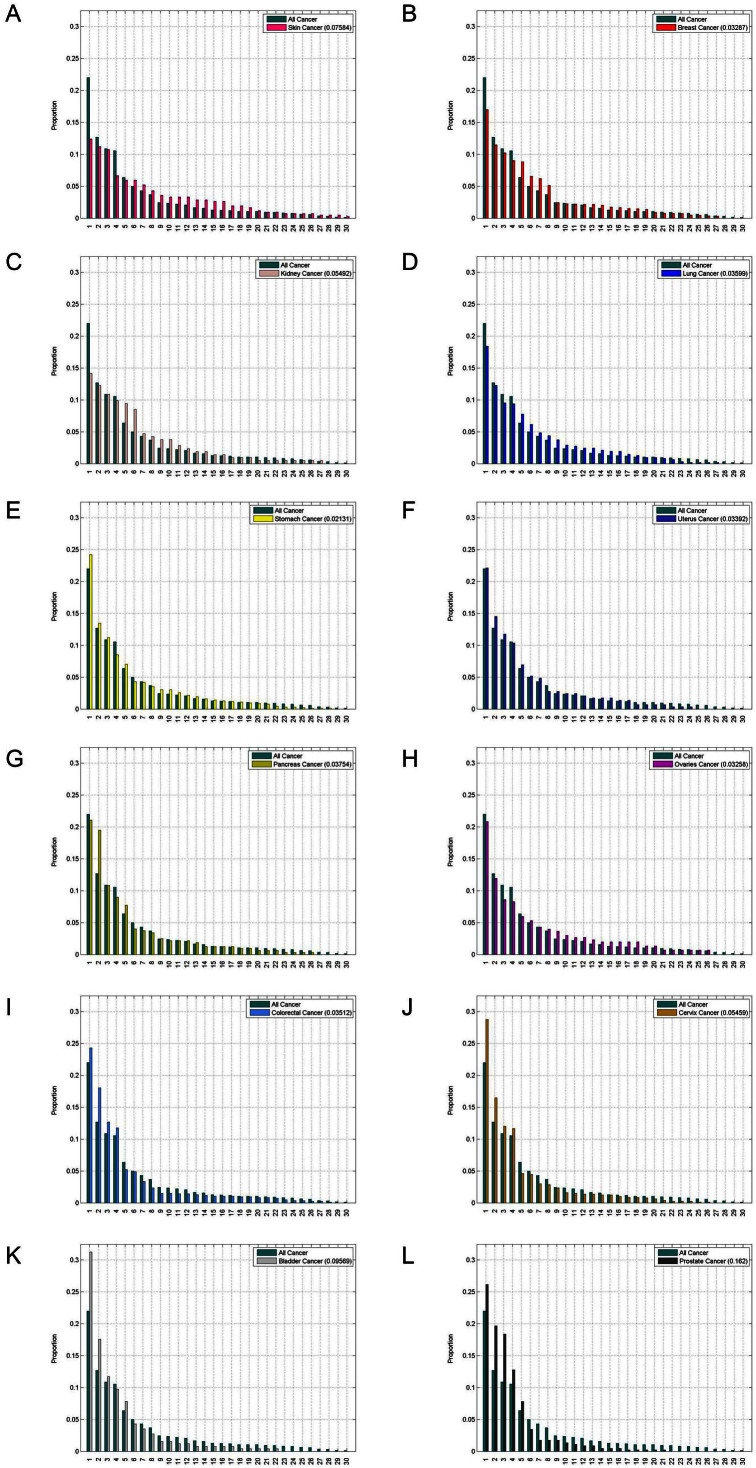
Histograms of distribution of metastatic tumors for primary cancers compared with distribution of all cancer. Data is plotted in descending order for each distribution, hence is not site specific. (a) Skin cancer; (b) Breast cancer; (c) Kidney cancer; (d) Lung cancer; (e) Stomach cancer; and (f) Uterine cancer. (g) Pancreatic cancer; (h) Ovarian cancer; (i) Colorectal cancer; (j) Cervical cancer; (k) Bladder cancer; and (l) Prostate cancer.

Column 6 in Table 1 shows the K-L computations between each of the cancer types and ‘All’ on a site specific basis, as shown in Figure 3. Here, we list the sites in decreasing order according to the all cancer category, meaning that the comparative histogram heights for each of the specific primary cancers generally are not arranged in strictly decreasing order. Thus, on this site-specific way of computing the K-L divergence, ovarian cancer (0.7995) and prostate cancer (0.2750) have the largest values, making them the most distinct from the ‘all’ cancer aggregate on a site-by-site comparison. By contrast, breast cancer (0.0759) and cervical cancer (0.0979) have the smallest values of site specific K-L divergence, meaning these are the most similar to the all cancer aggregate. In Figure 4, we show the same histograms as in Figure 3, but we arrange the sites in order of decreasing size. This way of comparing the distributions focuses on the shape of the distribution, i.e. the rate at which it drops to zero, rather than the actual sites to which the disease spreads.

### Markov diagrams, spreaders and sponges, and graph conductance

**Figure 5:**
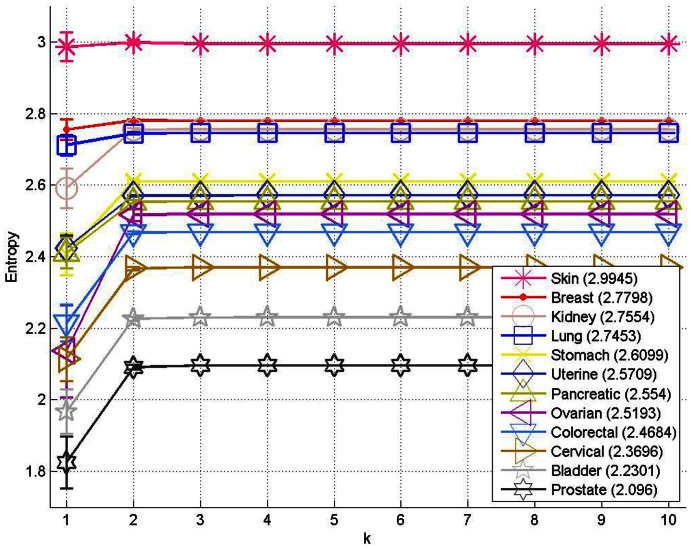
Metastatic entropy as a function of the model-based timescale, ‘k’. Entropy associated with the initial state vector (k = 0) begins at 0 and increases with each step over time.

The notion of entropy of metastatic tumor distributions is closely tied to systemic complexity of the disease, which in turn is tied to the fact that the metastatic process, both on the molecular level^24^,^25^,^26^,^27^,^29^ and across anatomical scales, is on average, an entropy increasing (or at least non-decreasing) dynamical process. The dynamics of progression from one anatomical site to the next can be captured reasonably well by modeling it as a Markovian process^28^,^30^ as it spreads from site to site. The state-vector, 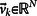 at discrete time-step *k* has entries that represent the distributed probabilities of a tumor developing at anatomical site ‘i’, 1 ≤ *i* ≤ *N*. The transition matrix 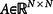 which is made up of probabilities of tumor spread from site ‘i’ to site ‘j’, propagates the state-vector forward in time via the Markov equations 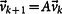. By using patient population data (this could be longitudinal or autopsy data), we obtain estimates of the model parameters, which are the transition probabilities that fill out the transition matrix *A*. See Materials and Methods section for details. Figure 5 shows the entropy for each cancer type, as the discrete model time-step, k, advances forward. For each of the cancers, the entropy values start at zero (at step k = 0, only the primary site has a tumor), then increase to their maximum value (corresponding to the steady-state) as the disease progresses, confirming that metastatic cancer is an entropy increasing process.

**Figure 6:**
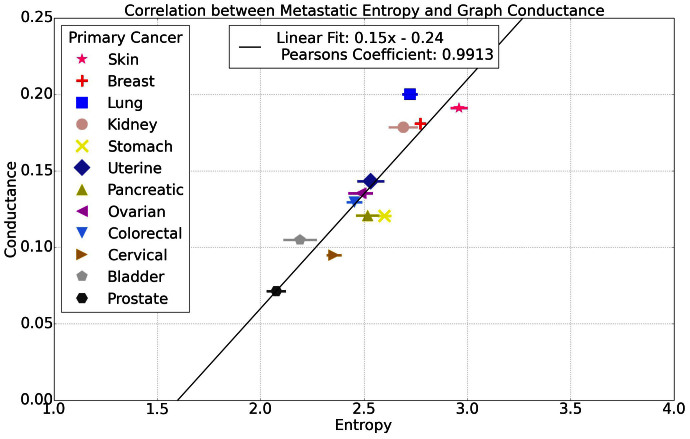
Graph conductance vs. entropy showing strong correlation across 12 cancer types.

The conductance is a measure of how fast a random walk converges to its steady-state distribution, which is tied to the Markov mixing time and convergence rate^30^. From the conductance values listed in Table 2, we can see a clear (although not perfectly one-to-one) correlation of entropy value and graph conductance value. The highest entropy and conductance cancers are skin, breast, kidney, and lung, the lowest in both are cervical, bladder, and prostate cancers. Table 2 summarizes the network based data for the 12 cancers. Note the overall decrease in graph conductance values listed in the 6^th^ column of the table, correlating roughly with decreasing entropy values for each. The correlation is more clearly shown in Figure 6 across all 12 cancer types.

**Figure 7:**
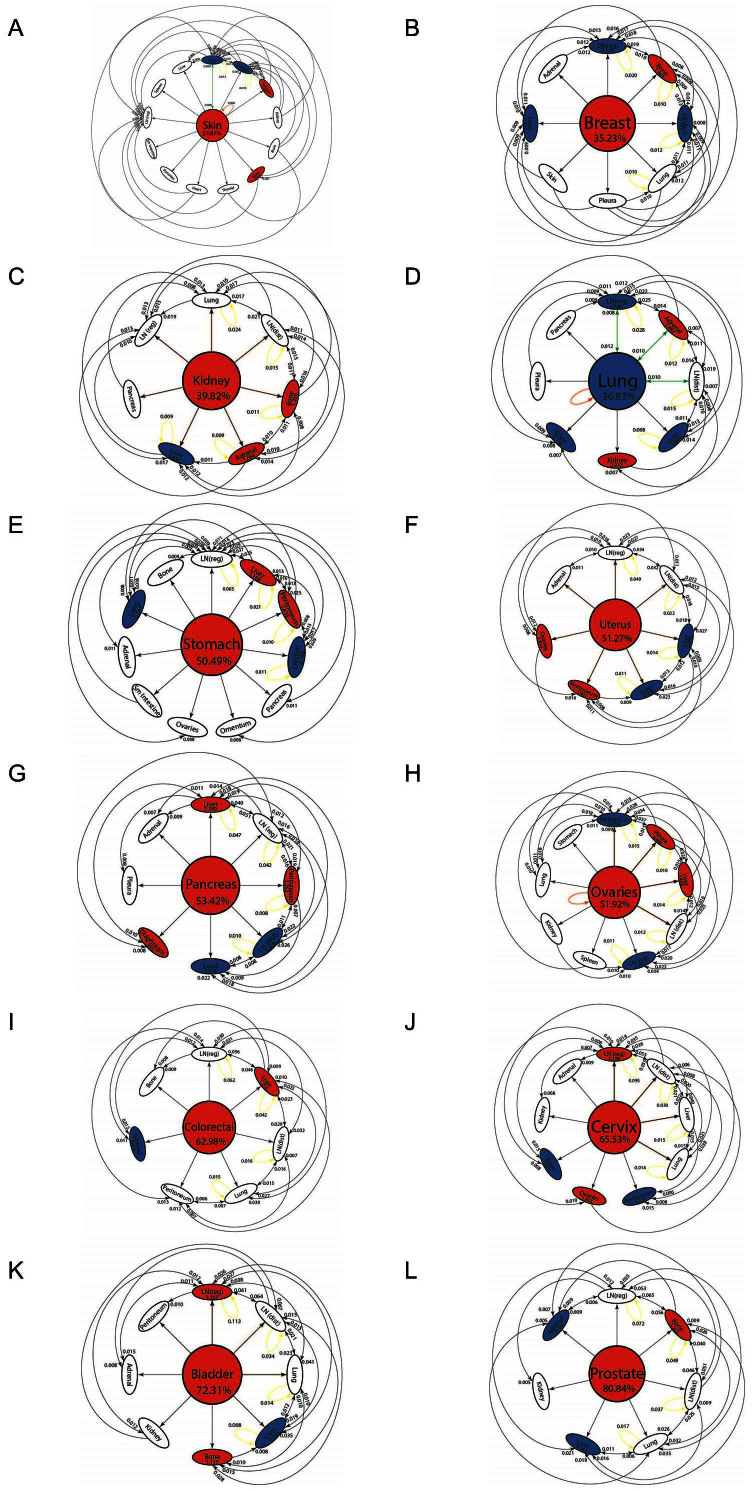
Top 30 two-step pathways emanating from primary tumors (total pathway probability listed in center node), obtained by multiplying the edges of the one-step edges comprising each two-step path. Edges without numbers are one-step paths. All other numbered edges mark the second edge in a two-step path, with numbers indicating the two-step probabilities. (a) Skin cancer; (b) Breast cancer; (c) Kidney cancer; (d) Lung cancer; (e) Stomach cancer; and (f) Uterine cancer. (g) Pancreatic cancer; (h) Ovarian cancer; (i) Colorectal cancer; (j) Cervical cancer; (k) Bladder cancer; and (l) Prostate cancer.

The reduced Markov diagrams for each of the 12 cancers, listed in the same order of decreasing entropy, are shown in Figure 7. These diagrams are based on retaining only the top 30 two-step pathways from primary site, to the first metastatic site, to the second metastatic site^17^,^18^. The % listed under the main primary circle represents the % that these 30 paths capture out of the total, hence is a separate measure of complexity of the cancer type. The top 30 paths associated with skin cancer (Figure 7a), for example, capture only 23.8% of the total, indicating that it has a more diversified set of alternative pathways available to it than, say, prostate cancer (Figure 7l), in which the top 30 pathways captures over 80% of the total. In order of decreasing entropy of each of the cancers whose reduced Markov diagrams are laid out from Figure 7a (highest entropy) down to Figure 7l (lowest entropy), the % of total pathways captured by the top 30 paths clearly increases (although, not exactly one-to-one), indicating that high entropy cancers have many alternative pathways and high graph conductance value (fast convergence rates to steady-state), whereas low entropy cancers have few pathways available, and low graph conductance (slower convergence rates to steady-state). Table 3 summarizes these metrics for the 12 cancer types, listed in order of decreasing entropy. Column 2 shows clearly how the percentages covered by the top 30 pathways increase with entropy, column 3 shows how the number of paths associated with a fixed % (in the case 35%) decreases with decreasing entropy.

Also summarized in this table are the main spreader and sponge metastatic sites^18^ associated with each tumor type. This notion, developed and used for lung cancer models in^18^ is based on a calculation of the probability out (*P_out_*) of each node in the directed graph as compared with the probability in (*P_in_*), obtained by adding up the edge weights of all the outward directed edges from a site compared with the inward directed edges. A node in which *P_out_* > *P_in_* is called a spreader site, whereas a node in which *P_out_* < *P_in_* is called a sponge site. From our lung cancer models and data, we know that the combined characteristics of the primary tumor and the first metastatic site to which it spreads is an important determining factor of the future course of disease progression, particularly if the first metastatic site is a spreader site associated with that cancer. At this point, for cancers other than lung, this is a graph based metric only that would need further clinical and biological correlates.

## Discussion

Grouping cancers according to their dynamical and probabilistic characteristics offers an alternative point of view from the classical approach of classifying according to site of origin. In our models, site of origin does play an important role in determining which transition matrix governs disease progression, and therefore a crucial role in determining which metastatic sites are spreaders and which are sponges and pinpointing the entropy values of each cancer. But grouping cancers according to metrics that are associated with key dynamical features such as entropy and graph conductance is a potentially enlightening way to think about similarities and differences between cancers based on their comparative metastatic potential. It provides a quantitative framework that could help guide clinical strategies whose end goals could be re-stated in language highlighting, for example, entropy reduction strategies and strategies that decrease mixing times, with clear ways of measuring and optimizing these quantities. Similarly, identifying therapeutic strategies that target metastatic spreaders, particularly in the oligometastatic setting^19^,^20^,^32^, might prove to be an area where these mathematical models could be of particular clinical value.

## Methods

### Summary of autopsy data set

We used the DiSibio and French^14^ data set of metastatic tumor distributions based on autopsy studies collected for 3827 untreated cadavers from 5 different cancer facilities in New England between 1914-1943. The data reflect 9484 distinct metastatic tumors distributed over 30 anatomical sites for all of the major tissue cancers. The data represent natural disease progression, which is useful, but we caution that brain metastases are under-represented in the data since examination of the intracranial contents at that time was not routinely performed. See also studies such as^21^ which focus on anatomical progression patterns. The data has been used in^17^,^18^,^31^ to develop a Markov chain model for lung cancer progression, where the autopsy data is used as the Markov chain steady-state, from which transition probabilities are calculated. In this paper, we directly characterize the data, shown in Figure 1 (All cancers) and Figure 2 (12 different primary cancers) in terms of their empirical distributions, which predominantly follow power-law form^15^. Other related work focusing on the development of dynamical models based on metastatic progression patterns includes references^18^,^21^,^22^,^23^. While notions of entropy have been used previously in the context of gene expression profiles and epidemiology^24^,^25^,^26^,^27^,^29^, we know of no previous work that uses these notions to characterize the complexity of large-scale progression patterns.

### Definition of entropy

The notion of entropy we use is from the field of information theory and statistical mechanics^7^,^8^,^9^,^10^. Given a probabilistic distribution of states 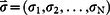 spread over N sites, the entropy associated with the distribution is given by the quantity 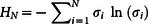 where 0 ≤ *H_N_* ≤ ln *N*. [To be clear, we are using the natural logarithm to define the entropy, hence the unit of measurement is commonly denoted as ‘nat’^8^. If base 2 logarithms were used, the units would be ‘bits’. One nat corresponds to 1.44 bits.] There are two factors that lead to increased entropy: (i) the larger the number N of sites over which the disease is distributed, the larger the entropy; (ii) the more even the probabilities are distributed among those sites, the larger the entropy. Thus, the lowest entropy state, given by *H_N_* = 0, corresponds to the distribution *σ_k_* = 1, *σ_i_* = 0 (*i* ≠ *k*)Since the probability of site ‘k’ being occupied is 1 and the probability of sites *i* ≠ *k* being occupied is 0, this state is associated with predictive certainty. In the language of statistical thermodynamics^10^, this would be called a completely ordered state. By contrast, the highest entropy state corresponds to the uniform distribution in which each site is equally probable, hence 
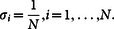
 This uniform distribution gives rise to a maximal entropy value of *H_N_* = ln(N) For this distribution, since each site is equally likely to occur with probability 1/N, the predictive certainty associated with this distribution is minimal, yielding the highest possible entropy value. We note that the entropy value is independent of the ordering of the sites. Thus, higher values of entropy are intimately tied to notions of disorder and complexity and have been used productively across a wide range of disciplines^6^,^7^,^8^,^9^,^10^,^11^,^12^,^13^.

### Definition of relative-entropy

The concept of relative entropy, or Kullback-Liebler distance, is used to measure the distance between two distributions of random variables^8^,^13^. One way to think of the relative entropy D(P||Q) between two random variables P and Q is to view D(P||Q) as a measure of *inefficiency* associated with assuming that the distribution is Q, when in fact the true distribution is P^8^,^24^.

It is defined as 
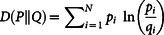
. In our comparisons, we use the symbol Q to represent the ‘All’ cancer empirical distribution, whereas P will represent a specific primary cancer type. Thus, the notion of relative entropy quantifies the relative inefficiency of using the generic ‘All’ cancer distribution instead of the more targeted and informative primary cancer type.

### Definition of graph conductance

Graph conductance is a measure of graph density, or how ‘well-knit’ the full graph is. The conductance is a measure of how fast a random walk converges to its steady-state distribution, which is tied to the Markov mixing time and convergence rates^30^. It is calculated for a network, *A*, by first partitioning *A* into two distinct sets 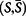 (where 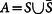 comprises all of the nodes in the network). The conductance associated with that particular partition is computed as the sum of the transition probabilities from all the sites in *S* to all those in 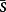, normalized by dividing by the sum of transition probabilities from sites in *S* or sites in 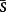 to ALL sites in the network 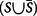, whichever of those two numbers is smaller. Then, the graph conductance is the minimum conductance achieved by calculating the conductance associated with all possible partitions of the network. More formally, it is defined as: 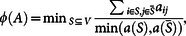
with
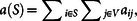
where *α_ij_* are the transition probabilities. Note that it is a quantity which uses the edge weightings of the directed graph, not just the adjacency matrix values.

### Construction of Markov transition matrices

The Markov transition matrices used in this paper are calculated using the same conditional random search algorithm used to construct the lung transition matrices described in^17^,^18^. Briefly, they are calculated using the following procedures:

1. The ‘target’ steady-state distribution for a given cancer type, denoted 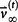, is defined as the right eigenvector 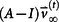 = 0, of transition matrix 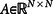, corresponding to unit eigenvalue. To construct the transition matrix for a given cancer type, we take the target steady-state distribution to correspond to the probability mass function associated with that cancer type from the data shown in Figure 2.
2. To construct the ‘final’ transition matrix for a given cancer type, *A_f_*, we construct a sequence of increasingly accurate approximations to *A_f_*, denoted (*A*_0_,*A*_1_,…,*A_i_*,…), with corresponding steady-state vectors 
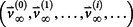
 so that 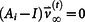 and 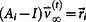, where 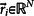 is called a residual vector, where 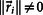, and 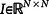 is the identity matrix. We condition our sequence based on an initial matrix *A*_0_, chosen so that the row associated with the primary tumor corresponds to the empirical distribution for that cancer type from the data, shown in Figure 2. All other rows are scaled so that they correspond to the empirical distributions associated with the appropriate cancer type, as shown in Figure 2. Note that this initial assumption cannot be the final converged transition matrix because it treats each metastatic tumor as a primary tumor. The iteration scheme then corrects for this quite efficiently in all 12 cases we considered, and finds the converged transition matrix which is nearest to this initial matrix. More details of this scheme and issues associated with uniqueness, robustness, and convergence are described for the case of lung cancer in Ref. 17.
3. To construct *A_i_*_+1_ from *A_i_* (the iteration procedure), starting with *A*_0_, we randomly perturb the entries of *A_i_*, in sequence from upper left to bottom right (see^17^ for more details), each time computing the corresponding residual 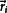. For each perturbation, if 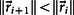, we keep the perturbation, if not, we discard it and perturb the next entry. The size of the perturbations are chosen to scale with the size of the residual vector, i.e. 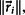 so that as we get closer and closer to converging to *A_f_*, the perturbations get smaller.
4. When 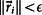, for a given convergence threshold value of *ϵ* (typically taken as O(10^−5^))), we stop the iteration and take the corresponding matrix *A_i_* as our converged transition matrix for that cancer type. We document the robustness and stability of the scheme by plotting the full set of singular values associated with an ensemble of Markov matrices produced this way, as well the one produced by averaging the ensemble – the method is robust, stable, and produces a unique Markov matrix with the correct steady-state for all 12 cancers studied in this paper.

## Figures and Tables

**Table 1:** Entropy table for each cancer type and for all cancers grouped together as one. First column lists the number of metastatic sites for that cancer type; second column lists the autopsy entropy value calculated from the original data; third column lists the sample entropy values as calculated from numerically produced sample populations using the Markov models and statistics from full population in data set (see text), fourth column lists the Kullback-Liebler divergence between that cancer type and the ‘all cancer' group, as compared in descending order for each; fifth column lists the K-L divergence between that cancer type and the all cancer group compared on a site specific basis. See text for more details

Primary Cancer Type	N	Autopsy Entropy	Sample Entropy	K-L Divergence	K-L Divergence (Site Specific)
Skin	30	2.9945	2.9598 ± 0.0406	0.0758	0.1373
Breast	27	2.7798	2.7737 ± 0.0180	0.0329	0.0759
Kidney	27	2.7554	2.6902 ± 0.0697	0.0549	0.1352
Lung	27	2.7454	2.7229 ± 0.0363	0.0360	0.1097
All	30	2.7136	2.7122 ± 0.0120	0.0000	0.0000
Stomach	28	2.6099	2.5996 ± 0.0314	0.0213	0.1191
Uterine	25	2.5709	2.5327 ± 0.0646	0.0339	0.1459
Pancreatic	27	2.5540	2.5177 ± 0.0555	0.0375	0.1392
Ovarian	22	2.5193	2.4838 ± 0.0588	0.9261	0.7995
Colorectal	29	2.4684	2.4532 ± 0.0368	0.3114	0.1271
Cervical	27	2.3696	2.3551 ± 0.0361	0.0546	0.0979
Bladder	23	2.2301	2.1910 ± 0.0806	0.0957	0.1477
Prostate	22	2.0960	2.0756 ± 0.0465	0.1620	0.2750

**Table 2:** Summary of network diagnostics

Primary Cancer Type	# of Nodes	# of Edges	Avg Edge Weight	Max Edge Weight	Conductance
Skin	30	900	0.03333	0.13115	0.1910
Breast	27	729	0.03704	0.17232	0.1808
Kidney	27	729	0.03704	0.17087	0.1784
Lung	27	729	0.03704	0.18794	0.2001
Stomach	28	784	0.03571	0.25834	0.1206
Uterine	25	576	0.04167	0.22161	0.1432
Pancreatic	27	676	0.03846	0.21103	0.1208
Ovarian	22	441	0.04762	0.24961	0.1353
Colorectal	29	784	0.03571	0.24418	0.1282
Cervical	27	676	0.03846	0.29013	0.0921
Bladder	23	484	0.04545	0.31865	0.1049
Prostate	22	441	0.04762	0.22627	0.0713

**Table 3:** Summary of reduced network diagnostics

Primary Cancer Type	30 Path % Value	35% Path Count	# of Sponges	Top Sponge	# of Spreaders	Top Spreader
Skin	23.81	54	2	Lung	2	Adrenal
Breast	35.23	30	3	LN (reg)	1	Bone
Kidney	39.82	25	2	Liver	2	Liver
Lung	36.83	28	3	LN (reg)	2	Adrenal
Stomach	50.49	14	2	LN (dist)	2	Liver
Uterine	51.27	15	2	Liver	2	Peritoneum
Pancreatic	53.42	12	2	LN (dist)	3	Liver
Ovarian	51.92	9	2	Peritoneal Cavity	2	Pleura
Colorectal	62.98	9	1	Adrenal	1	Liver
Cervical	65.53	7	2	Peritoneum	2	LN (reg)
Bladder	72.31	6	1	Liver	2	LN (reg)
Prostate	80.84	6	2	Liver	1	Bone
